# Role of Artificial Intelligence in Fighting Antimicrobial Resistance in Pediatrics

**DOI:** 10.3390/antibiotics9110767

**Published:** 2020-11-01

**Authors:** Umberto Fanelli, Marco Pappalardo, Vincenzo Chinè, Pierpacifico Gismondi, Cosimo Neglia, Alberto Argentiero, Adriana Calderaro, Andrea Prati, Susanna Esposito

**Affiliations:** 1 Pediatric Clinic, Pietro Barilla Children’s Hospital, Department of Medicine and Surgery, University of Parma, 43126 Parma, Italy; umbertaker@msn.com (U.F.); marco.pappalardo@studenti.unipr.it (M.P.); chinevincenzo@gmail.com (V.C.); pgismondi@ao.pr.it (P.G.); negliamino@gmail.com (C.N.); alberto.argentiero@unipr.it (A.A.); 2 Microbiology and Virology Unit, Department of Medicine and Surgery, University of Parma, 43126 Parma, Italy; adriana.calderaro@unipr.it; 3 Department of Engineering and Architecture, University of Parma, 43126 Parma, Italy; andrea.prati@unipr.it

**Keywords:** artificial intelligence, machine learning, neural networks, antimicrobial resistance, antimicrobial stewardship, pediatrics, children

## Abstract

Artificial intelligence (AI) is a field of science and engineering concerned with the computational understanding of what is commonly called intelligent behavior. AI is extremely useful in many human activities including medicine. The aim of our narrative review is to show the potential role of AI in fighting antimicrobial resistance in pediatric patients. We searched for PubMed articles published from April 2010 to April 2020 containing the keywords “artificial intelligence”, “machine learning”, “antimicrobial resistance”, “antimicrobial stewardship”, “pediatric”, and “children”, and we described the different strategies for the application of AI in these fields. Literature analysis showed that the applications of AI in health care are potentially endless, contributing to a reduction in the development time of new antimicrobial agents, greater diagnostic and therapeutic appropriateness, and, simultaneously, a reduction in costs. Most of the proposed AI solutions for medicine are not intended to replace the doctor’s opinion or expertise, but to provide a useful tool for easing their work. Considering pediatric infectious diseases, AI could play a primary role in fighting antibiotic resistance. In the pediatric field, a greater willingness to invest in this field could help antimicrobial stewardship reach levels of effectiveness that were unthinkable a few years ago.

## 1. Background

Artificial intelligence (AI) is an active and constantly evolving field of computer science research aiming to develop systems that simulate human intelligence and can perform tasks that normally require it such as visual perception, speech recognition, decision making, and natural language processing [1,2]. Crucial factors that have driven AI evolution are the availability of data from electronic health records (EHR) and advances in computational performance. These two factors are closely related to complex mathematical functions such as machine learning (ML) or neural networks (NN) [3]. This is even more relevant after the advent of deep neural network (DNN) architectures, where the complexity (often referred to the number of parameters the networks need to learn) has increased enormously in the last decade [4].

ML is a subset of AI that differs in its ability to change when presented with large amounts of data. While expert systems are manually-defined based on the expertise of humans, ML does not require (unless in some limited case) human intervention and try to automatically learn rules, similarly to how the human brain works. This property makes it less fragile and less dependent on human experts [5]. NNs, on the other hand, are mathematical informatics calculation models based on the functioning of biological neural networks (human or animal), in other words, models consisting of interconnections of information that are able to recognize the underlying relationships in a dataset. A DNN is composed of several layers (usually more than five) of processing units that allow us to improve predictions from the data, thus learning to understand them independently. A significant advantage of neural networks is that their performance improves progressively as the size of the dataset increases [4], adapting to changing inputs [6].

Currently, there are a large number of variables related to a patient’s care and medical history, which make patient management complex. A recent publication estimated that within the current year, 200 times more medical information than an individual would be able to read in their lifetime will be produced [7]. AI, by automatically managing this vast amount of data, can play a revolutionary role in supporting clinical decision making. However, even today, most doctors still do not understand the usefulness of AI and continue to make decisions based solely on personal experience and treatment guidelines [3]. The aim of our narrative review is to show the potential role of AI in fighting the growing phenomenon of antibiotic resistance, with particular reference to pediatric patients. For this reason, we searched for PubMed articles published from April 2010 to April 2020 containing the keywords “artificial intelligence”, “machine learning”, “antimicrobial resistance”, “antimicrobial stewardship”, “pediatric”, and “children”, and we described the different strategies for the application of AI in these fields. We considered this search period because it covers the vast majority of studies on the role of AI in infectious diseases. In this review, we focused on the use of AI for pediatric infectious diseases in developed countries.

## 2. History

In the 1960s, researchers at Stanford University developed the first problem solving program, “Dendral”, whose purpose was to evaluate hypotheses. It was to be used by pioneers in organic chemistry to identify unknown samples through their mass spectra. This first system was used to identify the bacteria causing serious blood infections and recommend appropriate antibiotic therapies [8]. However, the use of this system in clinical practice has been prevented by legal problems [9].

The use of AI in healthcare achieved popularity in 2016, when AI software integrated into the IBM Watson platform diagnosed a rare form of leukemia in a 60-year-old woman and proposed an effective treatment [10]. Regarding the pediatric field, in 1984, one of the first articles on the use of AI was published: it presented a computer-assisted medical decision making system called SHELP, which aimed at diagnosing inborn errors of metabolism [11]. Approximately 30 years later, the IBM Watson platform was successfully used at the Boston Children’s Hospital to provide valuable help in the diagnosis and treatment of rare pediatric diseases [12].

Since 1984, there has been a surge in publications on the use of AI in various fields of pediatrics: emergency management (automatic stratification of appendicitis risk [13], support for diagnostic decisions [14], and as a framework for asthma exacerbation and early prediction of hospital care [15]); pediatric oncology (comparative analysis of key genes for the development of anticancer drugs [16] and gene expression profiling of children with neuroblastoma or lymphoblastic leukemia [17]) and infantile neuropsychiatry (prediction of seizures in children with epilepsy [18], identification of motor abnormalities [19], and identification of children with autism spectrum disorder based on facial abnormalities [20]). In pediatric infectious diseases, there are many strategies for the application of AI, from the development of new antimicrobial drugs to the correct diagnosis and management of infectious diseases.

## 3. Antibiotic Resistance in Pediatrics

Antimicrobial resistance (AMR) is defined as the ability of a microorganism (bacterium, virus, or fungi) to prevent an antimicrobial agent from acting against it. AMR is considered a global public health emergency for both epidemiological and economic reasons to the extent that the World Health Organization has published an action plan on it. With regard to pediatrics, antibiotics are among the most widely prescribed drugs in children both in hospitals and in the community. However, there are many factors that can affect the use of these drugs. For example, many prescriptions are inappropriate or unnecessary: antibiotics are often used in children suffering from viral infections or even non-infectious diseases. In other cases, broad-spectrum antibiotics are prescribed at incorrect dosages or to treat infections for which targeted therapy is recommended. The emergence of multi-drug-resistant bacterial pathogens is thus strongly promoted, resulting in increased patient mortality, longer hospital stays, and higher health care costs [21].

Furthermore, the development of antibiotics is no longer considered an economically sensible investment for the pharmaceutical industry as antibiotics are used for relatively short periods of time, unlike drugs used to treat chronic diseases; the development costs of the latter are, among other things, much lower than those of antibiotics [22]. The result is that over the last 15 years, there have been significant deficiencies in the development and availability of new antibiotics to combat emerging resistance cases [21]. Implementation of containment strategies to address this rapidly growing problem, an effort called antimicrobial stewardship (AS), is therefore essential. These strategies have had a positive impact on adult patients, but only recently have they been used in the pediatric field [23], where, considering the heterogeneity in the age and weight of the patients, targeted interventions are needed [21]. As a consequence, the next section will report and discuss possible applications of artificial intelligence against AMR.

## 4. Application Strategies for Artificial Intelligence (AI) Against Antibiotic Resistance

### 4.1. Prediction, Assessment and Diagnosis of Pediatric Infectious Diseases

A key aspect of fighting antibiotic resistance is the early recognition of the infectious pathology, the distinction between pathologies on an infectious or non-infectious basis, and the proper management of complications. Children have higher infection rates than adults and often exhibit non-specific symptoms, which increases diagnostic uncertainty [21]. AI is, in this sense, a potentially powerful weapon.

In 2017, Komorowski et al. presented a tool based on reinforcement learning, in which a virtual agent learns a set of rules from a trial and error system to optimize them and maximize the expected performance [24]. This tool extracted an amount of patient data that exceeded the life experience of human physicians by many fold and learned the optimal treatment of sepsis by analyzing multiple decisions by clinicians. Its use has resulted in lower mortality in patients for whom the doctors’ actual decisions matched those of AI, showing the clinically reliable ability of this tool to customize sepsis treatment and assist doctors in making real-time decisions [24].

A trial conducted in a German pediatric tertiary intensive care unit aimed to distinguish and diagnose infectious sepsis from non-infectious forms of SIRS at an early stage based on the concept that the two entities are characterized by very similar symptoms [25]. To this end, a diagnostic model based on ML, specifically on a random forest approach, was developed, taking into account 44 variables available at the time of patient admission (baseline characteristics, clinical/laboratory parameters, and technical/medical support). The model allowed for early recognition of all sepsis cases, and a potential reduction of 30% in the use of antibiotics in patients with non-infectious SIRS was calculated [25]. A further pediatric study was presented in 2019 by Liang et al., in which 101.6 million data points from 567,498 outpatients were analyzed.

The primary diagnoses took into account 55 diagnostic codes that considered common pediatric diseases. Among the most frequently found diagnoses were acute upper respiratory tract infections, bronchitis, bronchopneumonia, and acute tonsillitis [26]. However, the system also showed strong performance in the diagnosis of potentially life-threatening conditions such as meningitis. The analysis was carried out using logistic regression classifiers to establish a hierarchical diagnostic system that achieved excellent performance in all organ systems and subsystems, demonstrating a high level of accuracy of the expected diagnoses compared to the initial diagnoses made by a medical examiner [26].

These studies show that ML-based applications can analyze EHR in a way similar to the hypothetical deductive reasoning used by physicians and could therefore be applied for purposes such as assessing triage procedures or assisting physicians in the diagnosis of complex or rare conditions. A relevant advantage is the reduction in inappropriate testing and cost.

### 4.2. Appropriate Prescription of Antibiotics

In general, appropriate prescription of antimicrobials is a complex challenge, as it involves selecting the appropriate therapy for the suspected pathogen, regulating the antimicrobial agent concentration and frequency of administration, and identifying the appropriate route to ensure that the actual drug levels reach the site of infection [27]. In the pediatric field, it must also be considered that the types of infection and resistance vary significantly with age, and there is a wide variability in dosage by age and weight [21].

One of the difficulties in prescribing antimicrobials is the need to sequentially adjust a patient’s treatment as new clinical data become available. The lack of specialized healthcare resources and the large amount of information to be processed make manual surveillance unsustainable; therefore, hospitals are increasingly relying on automated decision support systems for the review of antimicrobial prescriptions. Most prescription monitoring systems use a rule base acquired from published and expert guidelines to identify inappropriate prescriptions and prevent potential adverse events. These systems are often poorly defined and therefore generate a high rate of clinically unhelpful alerts.

To overcome this problem, systems based on ML have been developed. In 2014, Beaudoin et al. described APSS, an antimicrobial monitoring system that, unlike the previous systems, was able to learn new rules for prescription surveillance. This feature, combined with user feedback supervision, was designed to enable APSS to self-improve its knowledge base in the long term. APSS showed an indication learning capability that enabled an appropriate and clinically significant transition from intravenous to oral antimicrobial therapy [28]. Generally speaking, in the NN literature, it is well known that the so-called “static” NN systems (where the training of the NN is performed only once, at startup) are often unsuitable in certain applications where the knowledge base changes over time. Therefore, more appropriate learning strategies have been proposed such as reinforcement learning (as opposed to the previously described, referred to as supervised learning) or incremental online learning.

The same APSS system was used in a subsequent study to identify inappropriate prescribing practices not supported by local antimicrobial stewardship experts. The learning module was able to extract clinically relevant rules by identifying inappropriate prescriptions not recognized by the baseline system [27].

Starting from the assumption that the role of drug concentrations in the clinical outcomes of children with tuberculosis is not clear (probably due to differences in pathology trends between children and adults) and that the target concentrations for dose optimization are unknown, Swaminathan et al. used a set of AI algorithms including random forests (an ML technique implemented by aggregating the results of separated decision trees) to identify predictors of clinical outcome among 30 clinical, laboratory, and pharmacokinetic variables.

In this way, the researchers found that pharmacokinetic variability is probably an important factor in therapeutic failure and death in children with tuberculosis. They also identified drug concentration thresholds predictive of poor outcomes and found a negative interaction between isoniazid and its companion agents, pyrazinamide and rifampicin, within certain concentration ranges [29].

The problem of health care costs and staff shortages in the management of the appropriate antibiotic prescription is particularly important in developing countries. For this reason, in 2018, a group of researchers hypothesized that by applying machine learning approaches to readily collect patient data, it would be possible to obtain low-cost individualized predictions for targeted empirical choices of antibiotics.

Blood culture data collected from a 100-bed children’s hospital in north-western Cambodia between February 2013 and January 2016 were analyzed, and information on clinical characteristics, demographic characteristics, and living conditions was acquired using 35 independent variables, processed via machine learning algorithms to predict Gram stain results and whether bacterial agents could be treated with common empirical antibiotic regimens. The results showed that modern ML algorithms can amplify widely used logistic regression models by predicting antibiotic susceptibility. In this case, the random forest approach worked particularly well, especially for the prediction of resistance to ceftriaxone, the most widely used empirical antibiotic in patients in the study [30].

In addition, automated systems can play an important role in the real-time surveillance of the adverse effects of antibiotic therapies, thus contributing to their appropriate prescription. Kilbridge et al. showed the potential of these tools by implementing and evaluating an automated surveillance system modified to detect drug-related (including antibiotic-related) adverse events in pediatric patients. The study found that this type of system can be effective in detecting adverse effects in hospitalized children [31].

When used as part of a decision support system, the best approaches based on AI and, more recently, on ML should substantially increase the percentage of patients receiving effective empirical antibiotic therapy while minimizing the risks of increased resistance selection.

### 4.3. Predicting Antibiotic Resistance

An area where the use of AI is proving useful is in predicting antibiotic resistance; thus, it is a valuable aid to physicians in the care of their patients, considering that diagnostic tests and antibiotic resistance assays often require prolonged periods of time. Moreover, empirical therapy is more complex in the pediatric field than in adult medicine, as susceptibility differs with age [21]. Yelin et al. analyzed a 10-year longitudinal dataset of over 700,000 community-acquired urinary tract infections, identifying strong associations between resistance to six analyzed antibiotics and demographic characteristics, past urine culture results, and the history of drug purchases by patients. They then developed a personalized ML-based antibiotic resistance prediction model, which identified a higher peak risk in infancy and childhood for some antibiotics (e.g., nitrofurantoin) [32].

Using an antimicrobial susceptibility dataset from the Microbiology Laboratory of a third-level hospital in Greece, Feretzakis et al. proposed a methodology based on ML techniques by developing a model that could predict susceptibility to a specific antibiotic based solely on the source of the sample, the presumed site of infection, the Gram stain result of the pathogen, and previous susceptibility data. The precision achieved by the system was 72.6% (a performance that could be substantially improved by including patient clinical data), thus proving to be a potentially relevant aid to the physician [33].

A further method was developed to predict antibiotic resistance, although it operates through as-yet unrecognized paths, uses algorithms based on genomic information to predict the bacterial phenotype, and is enhanced by the continuous increase in the availability of high-density genomic data for a wide range of microorganisms. An example of this application is VAriant Mapping and Prediction of antibiotic resistance, a bioinformatics tool developed with machine learning techniques and with which sequencing data from 3393 bacterial isolates of nine species containing antibiotic resistance phenotypes for 29 antibiotics were analyzed. The researchers detected 14,615 variant genotypes and constructed 93 association and prediction models that confirmed the mechanisms of genetic resistance to known antibiotics, with an average accuracy of 91.1% for all antibiotic-pathogenic combinations [34].

A similar tool has been used to test over 10,000 isolates of M. tuberculosis collected from 16 countries on six continents, accurately predicting the point mutations associated with the emergence of antibiotic resistance to first-line drugs [35].

### 4.4. Artificial Intelligence and Pharmaceutical Industry

#### 4.4.1. Antimicrobial Peptides

Antimicrobial peptides (AMPs) are an example of functional natural biopolymers that are essential for all multicellular organisms and have evolved to cope with bacterial invasion and infection [36]. Described as ancient evolutionary weapons and found in both the animal and vegetable kingdoms, they play a fundamental role in the non-specific innate defense system that provides resistance to infection without prior exposure to foreign pathogens. Direct antimicrobial activity is not limited to membrane rupture mechanisms (through depolarization, the creation of pores that could cause the release of cell contents or alterations in the lipid composition in the double layer of the membrane [37]), but extends instead to cytoplasmic macromolecular synthesis, interference with membrane biosynthesis, and metabolic functions.

Our knowledge of natural antimicrobials dates back to the end of the 19th century, when studies on human phagocytes showed for the first time that some low molecular weight proteins were important for immunity. Bacteria have probably been exposed to AMPs for millions of years and, with the exception of some species (such as *Burkholderia* spp.), no widespread resistance has been reported, making AMPs potentially very effective. These bioactive peptides not only act as direct antimicrobial agents, but also represent important factors capable of strongly modulating the immune response through a number of activities including increasing the production and release of chemokines by immune and epithelial cells, exerting pro- and anti-apoptotic effects on different types of immune cells and stimulating angiogenesis. Moreover, AMPs have additional antimicrobial effects as they can suppress biofilm formation, stimulate chemotaxis, and mediate phagocytosis [38].

Currently, the most potent natural peptides known for their antimicrobial activity are the β hairpin peptides typified by polyphemusin I from the horseshoe crab. Most natural cationic peptides are much less active than synthesized peptides and are strongly antagonized by physiological concentrations of mono- and divalent cations as well as polyanionic polymers. Therefore, new approaches are needed to detect more effective and broad-spectrum, non-toxic sequences with optimal pharmacokinetics and selectivity profiles [38]. Here, AI can be useful.

Studies based on a model AMP with known activity seek to identify peptides with higher or lower antimicrobial activity; often via modification of a single amino acid within the peptide to identify amino acid positions important for activity, disregarding the interactions between amino acids that affect the overall three-dimensional conformation of the peptide [38]. On the contrary, AI models capable of modeling interactions between entities (e.g., amino acids) by means of recurrent NN or by graph-based architectures such as graph kernels or residue interaction networks, also known as residue interaction graphs, protein contact networks, or residue contact networks [38].

Biophysical modeling studies aim instead to understand the activity of AMPs through molecular dynamics simulations that may involve representation of the peptide, the surrounding solvent, and membrane portions; the model obtained is inevitably limited by the complexity of natural systems, since simplifications must be made in the computational approach [38].

Virtual screening, also called in silico screening, can be seen as a funnel approach in which one or more calculation methods are used to select a subset of compounds from a candidate molecule screen (usually in a molecular database) for experimental validation. The objective is to increase the probability of identifying the active compounds. Depending on the information available for the system, the search can be performed using either structure-based methods or ligand-based methods [39].

Among the examples of these applications is RiPPquest, a research database launched in 2014: starting from the extraction of the genome based on mass spectrometry through algorithmic tools, it has been able to discover a new natural antimicrobial peptide, informatipeptin from Streptomyces viridochromogenes [40].

In 2018, Yoshida et al. used methods based on artificial NN models to improve the antimicrobial activity of certain peptides against *Escherichia* *coli*. Starting from a natural AMP, they identified 44 highly potent peptides, and through conformational modification, they achieved an approximately 160-fold increase in antimicrobial activity [41].

There are promising possibilities for the use of antimicrobial peptides due to their ease of synthesis, mechanism of action, and broad spectrum of activity [38]. The use of AMPs in combination with antibiotics is another avenue to explore. In vitro results were found using the diastereomeric peptide against methicillin-resistant *S. aureus* and *P. aeruginosa*. The peptide showed synergistic or additive effects when used in combination with antibiotics [41].

However, there are still some limitations to consider: toxicity, difficulties of administration, high costs associated with large-scale production, and instability. Some concerns have also been expressed about the emergence of cross resistance—although considered rare—through a mechanism of bacterial membrane alteration [37].

#### 4.4.2. Discovery of New Antibiotics

The great power of neural network-based models in the development of antimicrobial drugs has recently been shown, thanks to a study conducted by Stokes et al. and published in 2020 [42]. These researchers developed a deep neural network model to predict growth inhibition of *E*. *coli* using a collection of 2335 molecules. Second, they applied the model to several chemical databases that included a total of >107 million molecules to identify potential active lead compounds against *E*. *coli*. After ranking the compounds according to the scores predicted by the model, they finally selected a list of candidates based on a predetermined predictive score threshold, the chemical structure and the availability. This process made it possible to identify a molecule, a c-Jun N-terminal kinase inhibitor renamed halicin, which was demonstrated to be a potent inhibitor of *E*. *coli* growth through mechanisms different from those of conventional antibiotics. In addition, halicin has been shown to be therapeutically effective against Clostridium difficile and pan-resistant Acinetobacter baumanii in mouse models.

Researchers detected eight antibacterial compounds in addition to halicin that were structurally distant from known antibiotics. In this way, they demonstrated how the use of AI can both decrease the cost of identifying guide molecules and increase the rate of identification of structurally novel compounds with the desired bioactivity in much less time than historically needed [42].

## 5. Limitations of Artificial Intelligence (AI)

AI has reached a level of accuracy in healthcare that was unimaginable until a few years ago, but there are numerous limitations that still make it difficult to translate into care pathways. First, the lack, especially in the pediatric field, of randomized clinical trials that demonstrate the reliability and/or improved effectiveness of AI systems compared to traditional systems in diagnosing infectious diseases or suggesting appropriate therapies creates a certain mistrust on the part of physicians toward the use of systems based on AI. The AI culture itself is lacking in health care personnel: many doctors—in our case, pediatricians—have never heard of AI [8].

Another limitation is the methodological bias that these systems may present as they are often based on studies, databases, and guidelines from other countries that may not be representative of all patients [43].

Another relevant limitation relies on the need for a large amount of data. Depending on the complexity of the AI/ML architecture used, the demand of data might increase (e.g., deep neural networks require a large amount of data). Even more, not only might the required data be numerous, but also their quality should be high, in terms of both data cleaning and data variability (NN and DNN are inclined to overfit data if the variability, e.g., in terms of variance, is limited). There are certain applications also within the pediatric field, where the collection of a large amount of clean, certified, and variable data might be hard, if not unfeasible.

Another topic concerns the protection of privacy and security: we can consider, for example, the need for consent to the processing of personal health data by artificial intelligence systems [44]. We must also think about how these systems should be integrated into the working environment of doctors and nurses. It may be necessary for these systems to provide the evidence behind their reasoning so that health professionals can decide whether to follow the suggestion.

Finally, ethical issues have to be considered such as the effects of de-qualification and desensitization of doctors to the clinical context, job losses [45], or more advanced and unpredictable scenarios: in 2017, for example, Facebook was forced to suspend an AI program because two systems began to communicate with each other using a language unknown to humans [46].

## 6. Conclusions

AI-driven health interventions fit into four categories relevant to global health researchers: (1) diagnosis, (2) patient morbidity or mortality risk assessment, (3) disease outbreak prediction and surveillance, and (4) health policy and planning [47]. A study conducted by IBM in 2017 estimated that 90% of the data currently available worldwide on AI were generated in the previous two years. In the healthcare sector, there has been a proliferation of sources generating these data; data are generated by genomic analyses, collected from medical apps and wearables used in healthcare contexts, and stored in databases containing medical records or retained in shared guidelines. This expansion of data has allowed, together with technological advances, a revolution in AI. In 2019, Peiffer et al. detected approximately 60 different ML applications that could be used in decision support for infectious disease management [48]. The applications in health care are potentially endless, and specifically considering infectious pediatric pathology, these systems could play a primary role in fighting antibiotic resistance, contributing to a reduction in the development time of new antimicrobial agents, greater diagnostic and therapeutic appropriateness, and, simultaneously, a reduction in economic and health personnel costs. In addition, AI can be used for health hygiene, infection control, and vaccination coverage at local, national, and international levels. Table 1 summarizes the application strategies for AI against antibiotic resistance.

Despite the field remaining nascent, AI-driven health interventions could lead to improved health outcomes in low and middle income countries due to its low cost under certain conditions. However, further studies are needed on ethical, regulatory, or practical considerations required for AI widespread use. The global health community will need to work quickly to establish guidelines for development, testing, and use as well as develop a user-driven research agenda to facilitate its equitable and ethical use.

## Figures and Tables

**Table 1:** Application strategies for artificial intelligence (AI) against antibiotic resistance.

AI Application in Fighting Antimicrobial Resistance	Definition	Advantages	Limitations
AI, health industry and antibiotics			
1. Antimicrobial peptides	Natural functional polymers, defensive elements for all multicellular organisms to counter bacterial invasion and infection.	- low risk of resistance development; - multiple antimicrobial mechanisms of action; - ease of synthesis thanks to AI.	- high toxicity to eukaryotic cells; - high cost of large-scale production; - initial appearance of cross resistance associated with widespread use; - onset of allergic reactions.
2. Discovery of new antibiotics	Discovery or development of antibacterial agents structurally different from known antibiotics.	- ability to develop new molecules with targeted and broad-spectrum bioactivity; - reduced time and labor costs for development.	- need for training libraries to contain molecules with physicochemical properties consistent with those of antibacterial drugs yet sufficiently diverse; - need for selection of the most appropriate approach compound development and minimizing toxicity.
AI, pediatric practice and infectious diseases			
Prediction of antibiotic resistance	Using machine learning (ML) techniques to predict the susceptibility of a microbial agent to an antibiotic.	- ability to exploit genomic information to predict the bacterial phenotype (VAMPr); - ability to help the clinician select the correct antibiotic.	- lack of complete genotypes in the NCBI database for each microorganism - need for integrating large amounts of data (laboratory, clinical, geographical)
Appropriate prescription of antibiotics	Selection of the appropriate therapy for the suspected agent, the appropriate dose and the correct route of administration.	- automated decision support systems for the review of antimicrobial prescriptions at hospital level; - ability to receive feedback for automatic and continuous improvement - guideline-based operation.	- lack of staff in systems management; - need for available health funds.
Prediction of infection severity	Automatic learning tools for the recognition of infectious pathology and correct management of complications.	- ability to distinguish infectious diseases, including sepsis, from non-infectious diseases - provision of decision support for the doctor; - ability to reduce mortality.	- need for accurate and complete data collection; - inability to obtain laboratory data from the beginning of illness.
